# The effect of early probiotic exposure on the preterm infant gut microbiome development

**DOI:** 10.1080/19490976.2021.1951113

**Published:** 2021-07-15

**Authors:** Yan Hui, Birgitte Smith, Martin Steen Mortensen, Lukasz Krych, Søren J. Sørensen, Gorm Greisen, Karen Angeliki Krogfelt, Dennis Sandris Nielsen

**Affiliations:** a Department of Food Science, Faculty of Science, University of Copenhagen, Frederiksberg, Denmark; b Department of Pediatrics, Hvidovre Hospital, Hvidovre, Denmark; c Department of Bacteria, Parasites and Fungi, Statens Serum Institut, Copenhagen, Copenhagen, Denmark; d Department of Biology, University of Copenhagen, Copenhagen, Denmark; e Department of Neonatology, Rigshospitalet,University of Copenhagen, Copenhagen, Denmark; f Department of Science and Environment, Roskilde University, Roskilde, Denmark

**Keywords:** Preterm, probiotics, gut microbiome, amplicon sequencing, necrotizing enterocolitis

## Abstract

Premature birth, especially if born before week 32 of gestation, is associated with increased risk of neonatal morbidity and mortality. Prophylactic use of probiotics has been suggested to protect preterm infants via supporting a healthy gut microbiota (GM) development, but the suggested strains and doses vary between studies. In this study, we profiled the GM of 5, 10 and 30-day fecal samples from two cohorts of preterm neonates (born <30 weeks of gestation) recruited in the same neonatal intensive care unit. One cohort (*n* = 165) was recruited from September 2006 to January 2009 before probiotics were introduced in the clinic. The second cohort (*n* = 87) was recruited from May 2010 to October 2011 after introducing *Lacticaseibacillus rhamnosus* GG and *Bifidobacterium animalis* ssp. *lactis* BB-12 supplementation policy. Through V3-V4 region 16S rRNA gene amplicon sequencing, a distinct increase of *L. rhamnosus* and *B. animalis* was found in the fecal samples of neonates supplemented with probiotics. During the first 30 days of life, the preterm GM went through similarly patterned progression of bacterial populations. *Staphylococcus* and *Weissella* dominated in early samples, but was gradually overtaken by *Veillonella, Enterococcus* and *Enterobacteriaceae*. Probiotic supplementation was associated with pronounced reduction of *Weissella, Veillonella* spp. and the opportunistic pathogen *Klebsiella*. Potential nosocomial pathogens *Citrobacter* and *Chryseobacterium* species also gradually phased out. In conclusion, probiotic supplementation to preterm neonates affected gut colonization by certain bacteria, but did not change the overall longitudinal bacterial progression in the neonatal period.

**Abbreviations:** GM: Gut microbiota; ASV: Amplicon sequence variant; NEC: Necrotizing enterocolitis; DOL: Days of life; NICU: Neonatal intensive care unit; ESPGHAN: European Society for Pediatric Gastroenterology, Hepatology and Nutrition; Db-RDA: Distance-based redundancy analysis; PERMANOVA: Permutational multivariate analysis of variance; ANCOM: Analysis of compositions of microbiomes; LGG: Lacticaseibacillus (former Lactobacillus) rhamnosus GG; BB-12: Bifidobacterium animalis ssp. lactis BB-12; DGGE: Denaturing Gradient Gel Electrophoresis

## Introduction

Preterm birth is defined as delivery before 37 weeks of gestational age^1^. During pregnancy the human organs develop in different phases.^2^ Premature neonates, with immature and fragile organs, usually encounter higher risks of morbidity and mortality, especially if born very preterm (before week 32 of gestation). Preterm birth complications was the leading cause of global neonatal deaths in 2018, accounting for approximately 35% of deaths among newborn infants.^3^ Necrotizing enterocolitis (NEC) is the most commonly acquired gastrointestinal morbidity among preterm neonates, especially those with very low birth weight (VLBW). Approximately 7% of VLBW infants develop NEC,^4^ and the 10–30% mortality rate unfortunately has not improved for years.^5^

With an aberrant gut microbiota (GM) compared to full-term infants,^6^ premature infants endure abrupt gut microbial population changes during the first 5–6 weeks after birth, where a schemed progression from *Bacilli* to *Gammaproteobacteria* and *Clostridia* takes place.^7^ The unstable GM as well as the immature gastrointestinal tract leaves these infants exposed to increased risk of e.g., NEC^8^ and sepsis^9^ in this period. These diseases are generally associated with GM dysbiosis. Although the proposed causative microbial agents differ between studies, *Enterobacteriaceae*-members such as *Klebsiella* spp. are often implicated.^10,11^

Probiotics, i.e., live microorganisms which confer a health benefit to host when ingested in adequate amounts, are regarded as one promising strategy to prevent neonatal NEC and sepsis.^12,13^ Probiotic supplementation to neonates born preterm has been proposed to prompt a GM resembling that of neonates born at term, enhance gut immunity and barrier, and therefore protect these vulnerable infants.^14–16^ Recently, the European Society for Pediatric Gastroenterology, Hepatology and Nutrition (ESPGHAN) has issued a conditional recommendation of probiotics for the prevention of NEC in preterm infants.^17^ A Cochrane meta-analysis based on 56 randomized, controlled trials (RCTs), including more than 10,000 children, also concluded that probiotic use might have promising potentials in reducing NEC and mortality cases among very preterm and very low birth weight infants, but due to the risk of bias in most trials, the authors called for further high-quality trials to inform the policy and practice.^18^ Probiotic effects are dose^12^ and likely strain specific.^19^ Similarly, also host specific factors influence e.g. probiotic colonization resistance in the gut.^20^ In one of the largest RCTs, PiPS,^21^ the administrated strain, *Bifidobacterium breve* BBG-001, showed no significant reduction of overall mortality and NEC among preterm neonates born before 28 weeks, but both highly varying colonization of the probiotic strain in the probiotic group and high cross-colonization in the placebo group were regarded as key factors affecting the primary end-points of the RCT. Re-analysis of the data indicated that neonates colonized by *B. breve* BBG-001 experienced clinically significant benefits for all outcomes when neonates shown to be colonized by the probiotic (detected in stool sample) was compared against neonates not colonized by the probiotic.^12^

*Lacticaseibacillus rhamnosus* GG (LGG) and *Bifidobacterium animalis* ssp. *lactis* BB-12 (BB-12) are well documented probiotics with beneficial effects on gastrointestinal tract and immune system.^14,22,23^ The prophylactic potentials of LGG and BB-12 have been confirmed in immature murine^24,25^ and piglet^23^ models and these strains in combination are among those recommended by ESPGHAN.^17^ Previously, we conducted one historically controlled cohort study of preterm neonates recruited before and after LGG and BB-12 were introduced as standard treatment for very preterm infants at one Danish neonatal intensive care unit (NICU).^26^ The clinical analysis indicated that supplementation with these probiotics was associated to a non-significant reduction in NEC grade 2–3 (OR = 0.78; 95% CI 0.43–1.39).^26^ To determine the influence of probiotic colonization on the GM, we profiled their fecal samples in the first 30 days of life by V3-V4 region 16S rRNA gene amplicon sequencing and linked this information to clinical phenotypic data.^27^

## Materials and methods

### Recruited infants and sample collection

Preterm neonates less than 30 weeks of gestation were recruited from one NICU at Rigshospitalet, Copenhagen, Denmark. From 2006 to 2009, neonates were recruited for the non-probiotic cohort (NoP cohort). The sampling and processing of stools was approved by the research ethics committee (KF 01 287895) and informed consent was obtained from the parents.

In March 2010, routine use of probiotics was implemented, using a commercial probiotic product, Bifiform® (Ferrosan A/S, Denmark), containing freeze-dried LGG (2 × 10^9^ CFU) and BB-12 (2 × 10^8^ CFU). The probiotic was added to 1 ml of mother’s breast milk or banked donor milk and given daily from day 3 of life. The clinical, historical study compared the all neonates at a gestational age < 30 weeks admitted to the NICU in two three-year periods and was registered at clinicaltrials.gov (NCT01670916).

To evaluate the probiotic effects on the GM, neonates were recruited for the probiotic cohort (PRO cohort) from April 2010 to Sept 2011, following the same feeding and treatment policy as the former cohort (NoP cohort), but supplemented with probiotics, as indicated above. The neonates’ individual information, feeding status and NEC diagnosis were collected. The oral nutrition ratio was determined as the percentage of the oral feeding as a percentage of the total fluid volume given enterally and intravenously. The clinical characteristics of included infants are summarized in the Supplementary Table 1.

**Figure 1.**
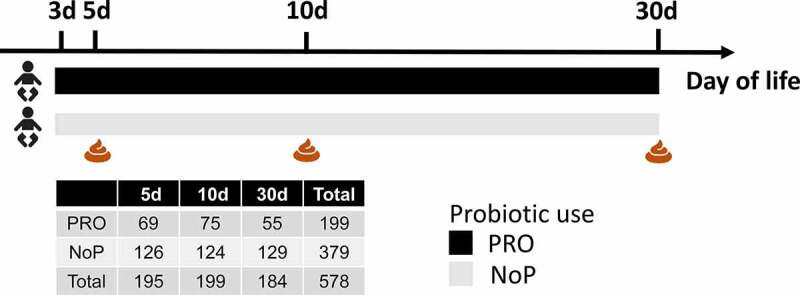
Graphical illustration of the study cohort. A total of 252 preterm neonates were included in this study, among which 87 neonates were given a probiotic mixture containing *Lacticaseibacillus rhamnosus* GG and *Bifidobacterium animalis* ssp. *lactis* BB-12 since the postnatal days of three. Fecal samples were collected at 5, 10 and 30 days of life. The adjacent table summarized the number of fecal samples from the PRO and NoP at three time points. PRO, probiotics cohort; NoP, non-probiotics cohort

Fecal samples were collected on postnatal days 3–5, 10, and 30 (Figure 1) by nurses from the infants’ diapers using sterile plastic spoons and put in sterile plastic screw-cap tubes.^27^ The samples were transferred to the microbiological laboratory at Statens Serum Institute for immediate preservation. Samples were mixed with 1 ml ox-broth and 10% v/v glycerol (SSI, Copenhagen, Denmark) and frozen at −80°C until further analysis.

### DNA extraction from fecal samples

DNA extraction from fecal material was performed according to published protocols.^28^ Briefly, 200 mg fecal matter were added to a 2 ml vial containing 1.4 ml ASL buffer (Qiagen, Stool lysis buffer). The sample was mixed with 0.3 g zirconium beads (diameter, 0.1 mm, Biospec Product Inc. Bartlesville, USA) and disrupted at 30 Hz for 6 min by a TissueLyser system (Qiagen Retsch GmbH, Germany). DNA was extracted by QIAamp DNA stool Mini Kit (Qiagen, Germany) according to the instructions by the manufacturer. DNA was eluted in a final volume of 100 µl and stored at −20°C until use.

### 16S rRNA gene amplicon sequencing

Amplicon sequencing was prepared with a two-step PCR method^29^ targeting 16S rRNA gene V3-V4 hypervariable region (Forward primer Uni341F: 5ʹ-CCTAYGGGRBGCASCAG-3ʹ, reverse primer Uni806R: 5ʹ-GGACTACNNGGGTATCTAAT-3ʹ).^30^ The PCR products were purified with Agencourt AMPure XP Beads (Beckman Coulter Genomics, 245 MA, USA), quantified with Quant-iT™ PicoGreen® quantification system (Life 246 Technologies, CA, USA) and pooled in equimolar concentration. The pooled libraries were paired-end sequenced (2 × 250 bp) on Illumina MiSeq System using MiSeq reagent kit V2. For each sequencing run, we also sequenced the negative controls in library preparation and one synthetic mock community containing equimolar rRNA from 20 bacterial strains (HM-276D, BEI Resources).

### Bioinformatic process pipeline

The bioinformatic analysis followed the standard procedures of Microbiome helper^31^ with minor changes. In short, the amplification primers were first removed with cutadapt (v2.4) and the trimmed reads were filtered for amplicon sequence variant (ASV) inference based on DADA2 (v1.12.1) algorithm.^32^ The error rate of sequencing was learned separately by run to infer the real biological sequence variants. Denoised reads were merged to construct a non-redundant ASV catalog after removing chimeric sequences using consensus mode of DADA2. The ASVs and abundance matrix were imported to QIIME 2^33^ (2019.07) for taxonomy assignment and phylogenetic tree alignment. The representative 99% similarity operational taxonomic unit sequences from Silva 132 rRNA database were used for training Naïve Bayes classifier on the exact amplified area using q2-feature-classifier.^34^ For lactobacilli, the genus-level taxonomic names were updated manually according to the recent announcement.^35^ The minimum frequency of ASVs was set as 0.1% of the mean sample depth to avoid MiSeq bleeding-through between runs.^31^ Besides, ASVs unclassified at phylum level and those classified as mitochondria and chloroplast were removed. Cross-contaminant ASVs in library preparation were identified independently in different batches with negative controls in default prevalence mode of decontam,^36^ resulting in the final ASV table. The repeatability of sequencing was inspected using mock samples from each run (Supplementary Figure 1). SEPP^37^ was used to align the remaining ASV sequences to construct a rooted phylogenetic tree. Finally PICRUSt2^38^ was used to infer the functional capacity of the GM based on the amplicon sequences.

### Quantitative estimation of ecological diversity

For the ecological analysis of the microbial community, samples were rarefied to even library size and samples with less than 1000 sequences were discarded (4.55% of all sequenced samples). The remaining 578 samples were used for following analysis by QIIME 2^34^ combined with R package phyloseq.^39^ Inverse Simpson, Shannon and Faith’s phylogenetic diversity were calculated to estimate alpha diversity while weighted and unweighted UniFrac dissimilarity metrics were adopted for beta diversity comparisons.

### Co-occurrence analysis

The neighborhood selection framework of SPIEC-EASI^40^ was adopted to estimate microbial association network in the neonatal gut. Samples were split into six groups based on days of life (DOL) period and probiotic use to find respective co-occurrence clusters. Core ASVs were chosen for calculation in each group under the threshold of mean relative abundance >0.1% and percentage of presence > 30%. The Meinshausen and Bühlmann (MB) neighborhood method was used to assess taxon-taxon interactions. The microbial clusters with MB coefficient >0.1 were visualized in a chord plot.

### Statistics

The statistical analysis was conducted in open-source statistical platform R^41^ (v3.6.2), except that alpha and beta diversity calculation and ANCOM^42^ were performed with QIIME 2^34^. R package compareGroups^43^ was used to analyze clinical information of the included infants. A *t*-test was used to determine the statistical difference for the continuous data i.e., gestational days, whilst Fisher’s exact test was used for categorical data of NEC incidence and mortality rate. For microbiome data, pairwise comparisons of alpha diversity were assessed by Wilcoxon rank-sum test with Bonferroni correction. The effect size of clinical covariates was estimated separately by the R^2^ from PERMANOVA (adonis function in R package vegan^44^). For each covariate, samples with missing information were removed for PERMANOVA test. Distance-based redundancy analysis (db-RDA) was performed to discern the variance explained by DOL period and probiotics use (based on weighted UniFrac distance metrics). ANCOM^42^ was applied to find the differentially abundant taxa between NoP and PRO and between different DOL periods. Wilcoxon rank-sum test with Bonferroni correction was adopted to determine functional capacity difference on the basis of PICRUSt2 output. R package ggplot2,^45^ circlize^46^ and complexheatmap^47^ were used to visualize the results.

## Results

### Cohort information and sample sequencing

In total, 252 preterm neonates were included (NoP vs. PRO, 165 vs. 87 neonates) for microbiome analysis, each neonate delivered at least one fecal sample profiled in adequate sequencing depth. Figure 1 presents a summary of the fecal samples analyzed in this study. The included infants were born after less than 30 weeks of gestation, with no statistical difference between the two groups (Supplementary Table 1). Among the included preterm neonates, we found distinctly decreased NEC incidence in PRO relative to NoP (3.45% vs. 13.3%). The all-cause mortality rate showed non-significant drop from 6.06% in NoP to 1.15% in PRO (*p* < .1). Importantly, the fecal samples analyzed in the present study were sampled during two larger prospective studies, where fecal samples were only collected during parts of these studies. When analyzing the entire two cohorts (also including infants, where fecal samples were not collected) it was found that supplementation with these probiotics was associated to a non-significant reduction in NEC grade 2–3 (OR = 0.78; 95% CI 0.43–1.39)^26^

### Probiotic use and postnatal age were two independent contributors to neonatal gut microbiota

**Figure 2.**
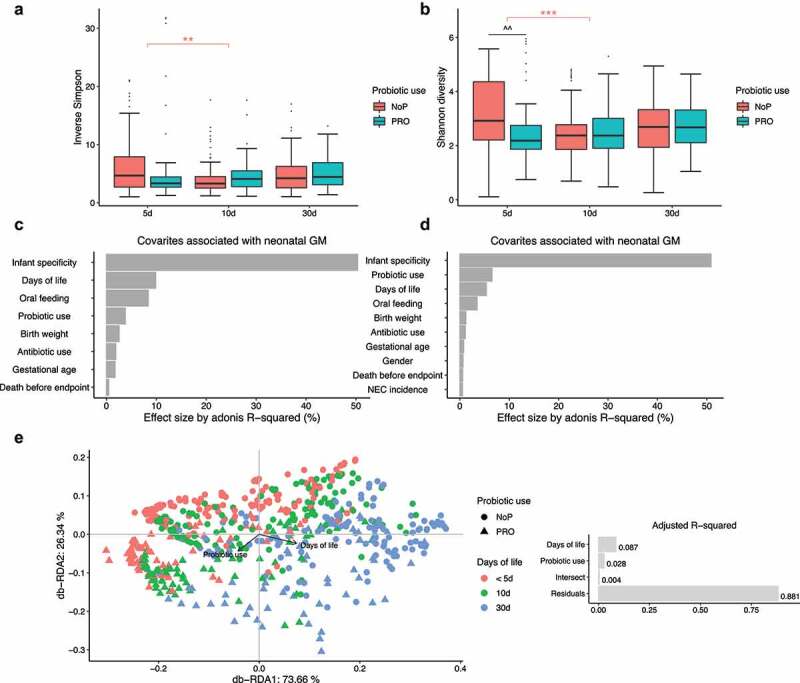
The effect of early probiotic use on the microbial diversity in the preterm gut. Inverse Simpson (a) and Shannon diversity index (b) of the gut microbial community, the effect size of gut microbiota-associated covariates determined in the weighted (c) and unweighted (d) UniFrac dissimilarity metrics, Db-RDA biplot showing microbial variance explained by probiotic use and DOL period (e), with adjusted R^2^ on the right panel. *, ** and *** represent adjusted *p* < .05, 0.01, 0.005 between different DOL periods, and its color indicates respective comparisons within PRO and NoP. ^ and ^^ represent adjusted *p* < .05, 0.01 between PRO and NoP in the same DOL period. PRO, probiotics cohort; NoP, non-probiotics cohort; DOL, days of life

The alpha diversity of the microbial community in the feces was estimated. NoP and PRO samples showed no significant difference as determined by the inverse Simpson index (Figure 2a). The Shannon diversity index which is more sensitive to rare species, PRO samples manifested a slight decline relative to NoP on day 5 (*p* < .01, Figure 2b). Both indices indicated no distinct longitudinal change of alpha diversity except for a decline on day 10 relative to day 5 in NoP (Figure 2a and 2b). Similar decreases were more apparent in the qualitative measures by ASV richness and Faith’s phylogenetic diversity (Supplementary Figure 2). As both metrics do not assign weights according to the ASV abundance, the declines in 5 and 10 day-of-life PRO samples, suggested probiotic exposure was related to the reduction of rare species. Using both weighted and unweighted UniFrac distance metrics (Figure 2c and 2d), the early-life GM showed substantial heterogeneity between individuals, which explained approximately 50% of variance, as determined by PERMANOVA. Probiotics use, DOL and oral nutritional ratio were the three main contributors besides the intra-individual difference. Probiotics showed larger effect size on the unweighted UniFrac distance metric (sensitive to rare lineages) relative to the other two, whilst oral nutrition and DOL contributed more to the weighted UniFrac dissimilarity. As expected, the oral nutrition ratio increased with postnatal age when we checked its distribution in PRO (Supplementary Figure 3). Although these data were only recorded for neonates in PRO, the collinearity of the two covariates corresponded to the comparable effect size on GM. Using db-RDA, we visualized the variance explained by probiotic use and DOL period (Figure 2e). The two contributors explained nearly 12% of total variance with no significant intersect effect. DOL period independently explained 8.7% of variance relative to 2.8% explained by probiotic use. The NEC cases explained only 0.5% of the GM variation, suggesting diverse microbial patterns among these samples (Figure 2d).

**Figure 3.**
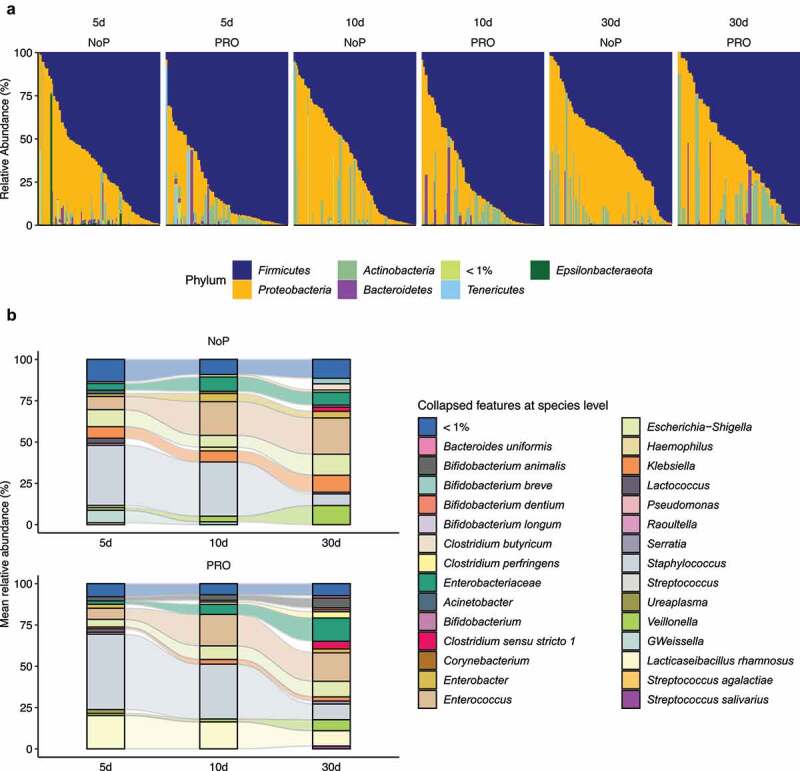
Summarized phylum- (a) and species-level (b) gut microbiota composition of PRO and NoP neonates. Prokaryotes with mean relative abundance below 1% are labeled as “< 1%”. PRO, probiotics cohort; NoP, non-probiotics cohort

### Early probiotic exposure did not alter the choreographed progression of the preterm gut microbiota

**Figure 4.**
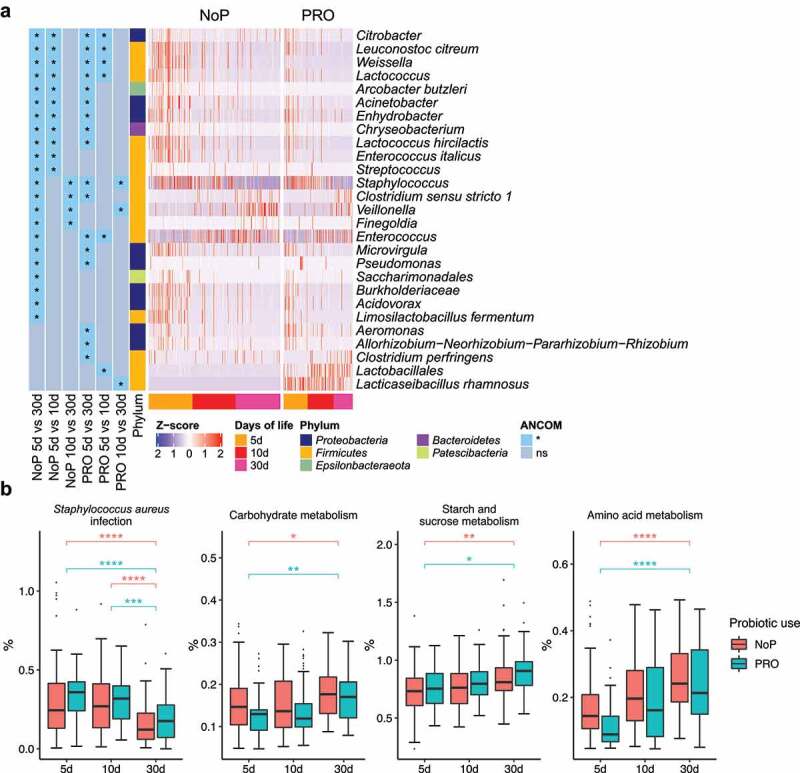
The longitudinal structural and functional progression of preterm gut microbiota. Differentially abundant taxa (a) and microbial functional capacities (b) between different DOL periods. Differentially abundant taxa were determined by ANCOM and labeled with * in the heatmap, indicating the significantly altered taxa from the respective between-group comparison. Row-wise Z-score scaling was conducted in the heatmap visualization, showing the normalized relative abundance by the mean of the specific taxa across all samples. In the boxplots, *, **, *** and **** represent adjusted *p* < .05, 0.01, 0.005, 0.001 between different DOL periods, and its color indicates respective comparisons within PRO and NoP. PRO, probiotics cohort; NoP, non-probiotics cohort

The most common phyla detected in these neonates’ GM were *Firmicutes, Proteobacteria* and *Actinobacteria* (Figure 3a). *Enterobacteriaceae-*related genera as *Klebsiella*, but also genera of *Enterococcus, Staphylococcus, Veillonella* and *Weissella* predominated the preterm GM (Figure 3b). The neonates in the PRO were characterized by a GM with higher relative abundance of *Firmicutes* and *Actinobacteria* due to the administrated *B. animalis* and *L. rhamnosus*. Regardless of the probiotic supplementation, *Enterococcus* and *Staphylococcus* were highly abundant, but the relative abundance of *Klebsiella, Veillonella* and *Weissella* were lowered among infants within PRO (Figure 3b). We used ANCOM to identify differentially abundant taxa in GM development, and found similar progression pattern in both groups (Figure 4a). *Proteobacteria* like *Citrobacter, Acinetobacter, Pseudomonas* were highly abundant on day 5 and declined quickly afterward. *Firmicutes* members varied in abundance with time. *Enterococcus, Clostridium* sensu stricto 1, and *Veillonella* became more prevalent with age and substituted some *Firmicutes* abundant initially, e.g., *Leuconostoc citreum* and *Weissella* spp. The relative abundance of the administrated probiotics fluctuated during the administration period. The relative abundance of *L. rhamnosus* declined on day 30 whilst the relative abundance of *B. animalis* remained constant during the observation period.

Using information from ASV-level amplicons, the functional capacity of the neonatal GM was predicted by PICRUSt2. Both NoP and PRO displayed patterned functional progression in the neonatal period (Supplementary Figure 4). Possibly attributed to increasing oral feeding ratio, the neonatal GM showed enhanced capacity of degrading carbohydrates and amino acids with age (Figure 4b). However, the gut inhabitants in the very early period (DOL < 5d), were enriched in genes encoding bacterial virulence e.g., *Staphylococcus aureus* infection. In line with this, we found the majority of predicted drug-resistance modules e.g., efflux pumps NorB and AbcA were enriched (Supplementary Figure 5), which corresponded to the enriched relative abundance of opportunistic pathogens, e.g., *Staphylococcus* and *Actinobacteria* spp. in this period.

**Figure 5.**
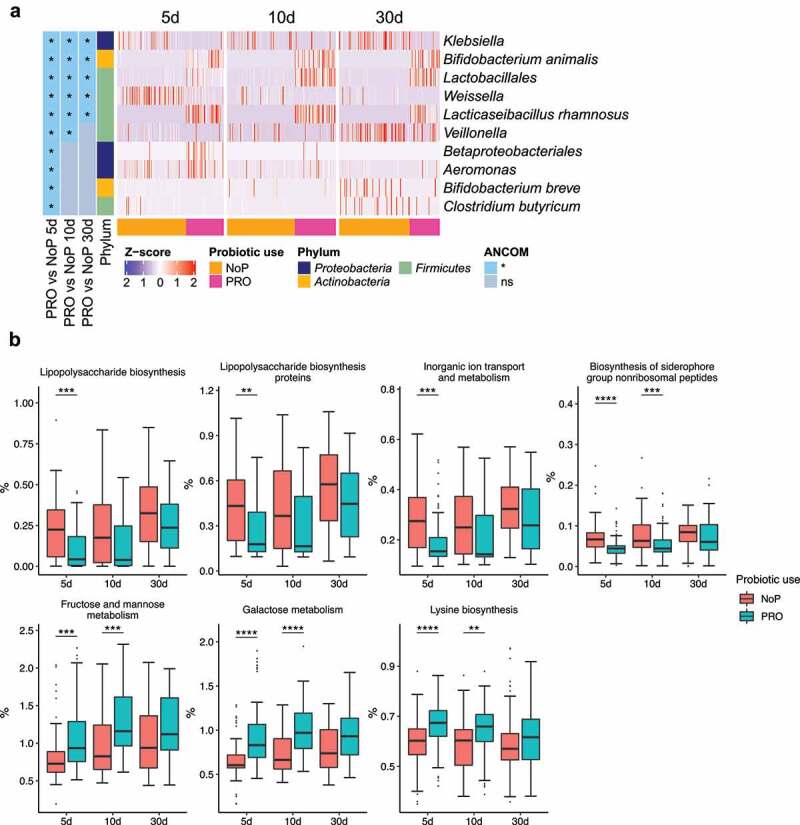
The gut microbiota compositional and functional shifts induced by probiotic exposure. Differentially abundant taxa (a) and functional capacities (b) between PRO and NoP. Differentially abundant taxa were determined by ANCOM and labeled with * in the heatmap, indicating the significantly altered taxa from the respective between-group comparison. Row-wise Z-score scaling was conducted in the heatmap visualization, showing the normalized relative abundance by the mean of the specific taxa across all samples. In the boxplots, *, **, ***and **** represent adjusted *p* < .05, 0.01, 0.005, 0.001 between PRO and NoP in the same DOL period. PRO, probiotics cohort; NoP, non-probiotics cohort

### Early probiotic exposure affected colonization by Klebsiella

Through 16S rRNA gene amplicon sequencing, we barely detected *L. rhamnosus* and *B. animalis* (i.e., the species to which the two probiotics belong) in the NoP samples (Supplementary Table 2). The two species were detected in most samples from the PRO but their relative abundance varied between samples. With a relative abundance threshold of > 0.1%, approximately 70% PRO samples were colonized by *B. animalis* and nearly 90% were colonized by *L. rhamnosus* (Supplementary Table 2). The relative abundance of *Klebsiella* and *Weissella* was significantly decreased in PRO at all three sampling points (Figure 5a). *Veillonella* experienced a stunted increase in PRO and its abundance did not reach a level comparable to NoP until day 30. The abundance of *Betaproteobacteria, Aeromonas* spp., *Bifidobacterium breve* and *Clostridium butyricum* only differed between the two groups on day 5. PICRUSt2 prediction also suggested a distinct change of GM functional capacity by probiotic use on day 5 and 10 (Supplementary Figure 6). Probiotic supplementation resulted in a GM with enriched capacity of fructose, mannose and galactose metabolism, and lysine biosynthesis, etc., but distinctly depleted bacteria possibly conferring virulence-relevant genes e.g., lipopolysaccharides (LPS) and siderophore biosynthesis, inorganic ion transport and metabolism (Figure 5b).

**Figure 6.**
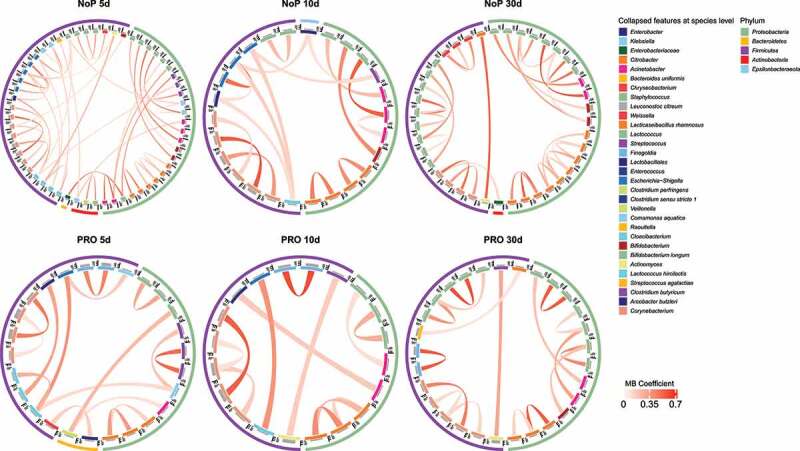
Gut microbiota co-occurrence network of PRO and NoP neonates in the first postnatal month. Core ASVs are used to calculate each sub-network under a threshold of relative abundance > 0.1% and percentage of presence > 30%. Taxon-taxon interactions were assessed by the Meinshausen and Bühlmann (MB) neighborhood method and the MB coefficients between taxa determine the color depth of inner chord graphs. The phylum-level information and the lowest taxonomic annotations are differentiated by color in the first and third circles respectively, and the mean relative abundance of taxa is shown by bar-plot in the second circle. PRO, probiotics cohort; NoP, non-probiotics cohort

### Preterm gut microbiota displayed loosely connected co-occurrence network*s* in the neonatal period

Co-occurrence of ASVs was calculated by SPIEC-EASI in the three separate DOL periods (Figure 6). The phylogenetic lineage was the primary factor reflected by the co-occurrence pattern. ASVs with the same taxonomy labels usually clustered together and the co-occurring microbes mostly came from the same phylum. Cross-phylum clusters were seldom seen. Most microbes like *Klebsiella* spp. had relatively low coefficients to others indicating a relatively isolated network architecture in terms of the direct relationship between haplotypes. We also seldom found co-occurring pairs for the two administrated probiotics in all DOL periods.

## Discussion

The neonatal GM is established from birth and evolve during the first 3 years of life before it is considered mature.^48^ Apart from the fact that a virtually sterile gut has to establish and stabilize a GM with a more or less random “seeding” from the mother, other caretakers and the environment, preterm neonates are colonized by an abnormal GM attributed to undeveloped organ and immune system due to shortened pregnancy length. Meanwhile they will go through different phases of GM development relative to the term neonates, which are influenced by longer hospital stays, delayed maturation of organs, etc. Here we utilized two preterm cohorts recruited before and after probiotic supplementation became the routine treatment in one Danish NICU, and investigated the GM development of these neonates in the first 30 days of life. Previously the samples of the present study have been analyzed by Denaturing Gradient Gel Electrophoresis (DGGE).^49^ DGGE showed a clear separation between NoP and PRO samples as also observed in the present study. Further, it was found that PRO samples resulted in more DGGE bands (a proxy measurement for observed species) than NoP. This is contrary to the present study, where more observed ASVs were observed in the NoP samples relative to PRO on day 5 and 10. The differences are likely due to the experimental differences including varying choices of primer (16S rRNA gene V3-V4 region in the present study, 16S rRNA gene V2-V3 region in the DGGE experiment^49^) and analysis platform (DGGE vs. high throughput amplicon sequencing). Using 16S rRNA gene amplicon sequencing, we found that *L. rhamnosus* and *B. animalis* colonized most neonates supplemented by LGG and BB-12. This might be what caused the reduced abundance of opportunistic pathogens e.g., *Klebsiella* spp. and *Clostridium butyricum*. In a recent large-scale metagenomic study, NEC was found to be preceded by increased bacterial replication and *Klebsiella* abundance in the fecal samples of preterm neonates,^10^ underlining that repressing *Klebsiella* likely provided a protection for the neonates. Toxigenic *Clostridium butyricum* strains have been reported to be implicated in pathological conditions of NEC as well.^50^ Also the relative abundance of this bacterial species was reduced among neonates in PRO. Interestingly, early probiotic exposure resulted in depletion of LPS- and siderophore-producing bacteria when we predicted the microbial functional capacity based on their ASV-level haplotype information. LPS^51^ and siderophores^52^ are virulence factors in many bacterial pathogens, and also LPS is implicated in the NEC pathogenesis.^53^ Recently, an observational study on preterm neonates born < 32 weeks of gestation, has also indicated probiotic supplementation could affect the colonization by *Escherichia, Enterococcus* and *Klebsiella*, leading to reduced NEC incidence.^54^ Similarly we found a reduced risk of NEC among the included infants (delivering fecal samples) in PRO relative to NoP. Although these findings indicated that probiotic supplementation resulted in the improved pathogen resistance of the neonatal GM and lowered NEC risk, this historically controlled study lacked the blinding of treatment allocation and the single-center design may lose generalizability to a broader population. Besides, the reduced NEC risk in PRO was not statistically validated in the original complete cohort, suggesting other underlying factors, e.g., drug use and host differences may confound the efficiency of probiotic supplementation.

During the first 30 days of life, the preterm GM is highly dynamic. Strongly connected microbial networks in the gut were not found among these neonates. Most co-occurring haplotype clusters e.g., *Klebsiella* spp. came from the same taxonomic assignments and seldom co-occurred with the others. Similarly, neither of the two probiotic species joined the co-occurrence microbial network. This suggests that the administrated probiotics influence the GM by occupying niches, utilizing resources available for some bacteria e.g., *Klebsiella*, but not positively interacting with other GM members.

The probiotic exposure altered the colonization by certain bacterial species, but it did not affect the longitudinal GM progression. The preterm GM manifested a patterned development trajectory in the neonatal period. We found that genera *Veillonella, Enterococcus* and the family of *Enterobacteriaceae* gradually overtook the previously dominant *Staphylococcus* and *Weissella* species during the first weeks of life. More *Bifidobacterium* and *Clostridium* appeared by the end of the first month after birth. It is believed that the high gut redox potential leads the preterm GM to have prolonged colonization of facultative anaerobes e.g. *Enterobacter, Enterococcus* and *Staphylococcus*, with delayed presence of obligate anaerobes e.g. *Bifidobacterium* and *Clostridium*.^55^ From our data, facultative anaerobes e.g., *Enterococcus* and *Staphylococcus* occupied the preterm gut in the first month of life. Species associated with nosocomial infections e.g., *Citrobacter, Pseudomonas* and *Chryseobacterium* spp. appeared in early collected samples (DOL ≤ 5d) but disappeared with age in both cohorts. Given the low oral feeding ratio and prevalent medical contact, the NICU environment^56,57^ may serve as a key source for the gut colonists in this period. PICRUSt2 results also confirm that the very early life GM showed increased presence of predicted drug resistance genes, which might be associated with common use of antibiotics in this population. Although unfortunately, we did not have re-access to the complete medical records to support this.

Oral feeding is another important factor in shaping the neonatal gut, and might explain the similarly patterned GM development between NoP and PRO. The neonates in both cohorts were solely provided with mother’s breast milk or donor bank milk. The records in PRO indicated that the oral feeding ratio increased with age as expected. This agrees with the comparable effect size on GM development, and the presence of *Bifidobacterium, Clostridium* and *Bacteroides* in the later periods. Although the feeding information was not recorded in NoP, similarly patterned GM progression were found between the two cohorts. The controlled feeding policy ensured the similar nutritional intake for the infants. The provided mother’s/bank milk contained bioactive compounds e.g., secretory IgA^58^ and human milk oligosaccharides^59^ and the commensal microbes,^60^ and these together contributed to the GM progression. Microbial functional prediction also suggested an interesting shift toward improved capacity to digest carbohydrate and amino acids when the neonates grew older and received higher ratio of oral feeding. These findings are in correspondence with the accepted theory that human milk could benefit the GM development, suppress the overgrowth of opportunistic pathogens e.g., *Staphylococcus* and *Pseudomonas*, and reduce the NEC risk among preterm neonates.^61,62^

## Conclusion

Early supplementation of LGG and BB-12 could colonize the preterm gut and affect potential pathogen (e.g., *Klebsiella* spp.) colonization. The neonatal GM manifested similarly schemed development trajectory regardless of probiotic use.

## Supplementary Material

Supplemental Material

## Data Availability

According to the general data protection regulation rules in Denmark, the raw sequencing data and analytic codes are available upon request.
