# Supplementary material for: The effect of early probiotic exposure on the preterm infant gut microbiome development
Source: Gut Microbes. 2021 Jul 15;13(1):1951113. doi: 10.1080/19490976.2021.1951113 (PMC8284123; doi:10.1080/19490976.2021.1951113)
Supplement: Supplemental Material [file KGMI_A_1951113_SM3485.zip › supplementary/downloadFromZipFile1.pdf]

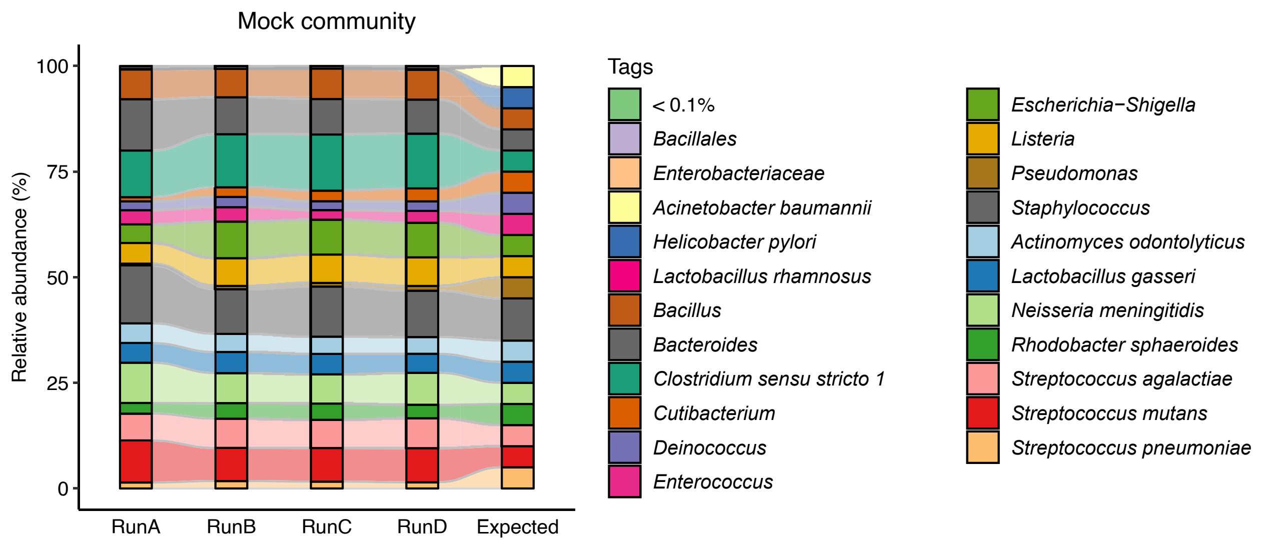

Mock community
Relative abundance (%)
100
75
50
25
0
RunA
RunB
RunC
RunD
Expected
Tags
< 0.1%
Bacillales
Enterobacteriaceae
Acinetobacter baumannii
Helicobacter pylori
Lactobacillus rhamnosus
Bacillus
Bacteroides
Clostridium sensu stricto 1
Cutibacterium
Deinococcus
Enterococcus
Escherichia−Shigella
Listeria
Pseudomonas
Staphylococcus
Actinomyces odontolyticus
Lactobacillus gasseri
Neisseria meningitidis
Rhodobacter sphaeroides
Streptococcus agalactiae
Streptococcus mutans
Streptococcus pneumoniae
