# Supplementary figures and images for: The effect of early probiotic exposure on the preterm infant gut microbiome development
Source: Gut Microbes. 2021 Jul 15;13(1):1951113. doi: 10.1080/19490976.2021.1951113 (PMC8284123; doi:10.1080/19490976.2021.1951113)

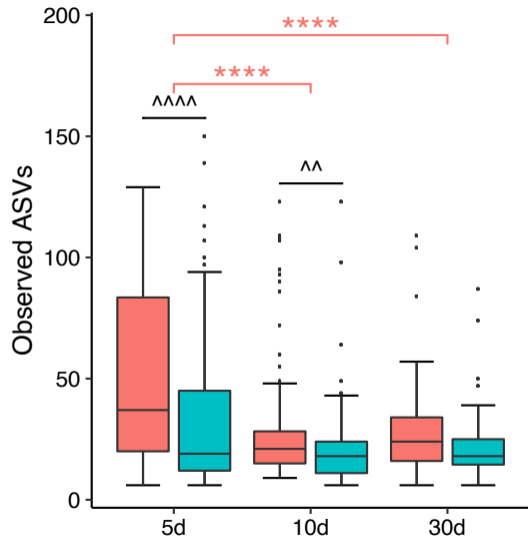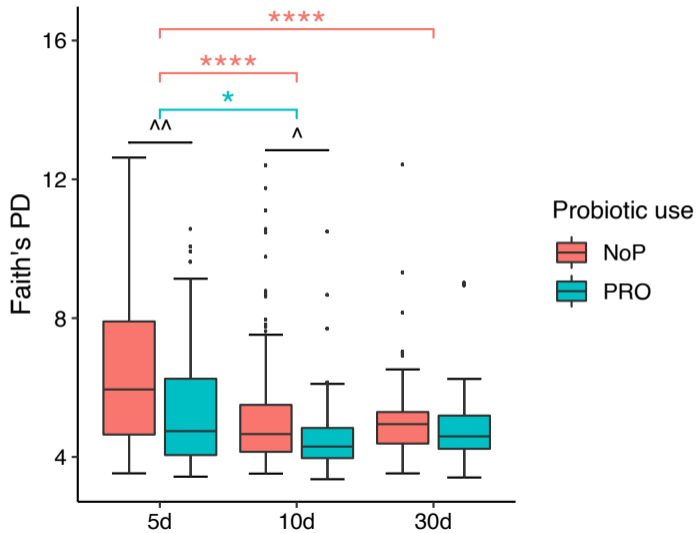

Supplement: Supplemental Material [file KGMI_A_1951113_SM3485.zip › supplementary/downloadFromZipFile2.pdf]

5d

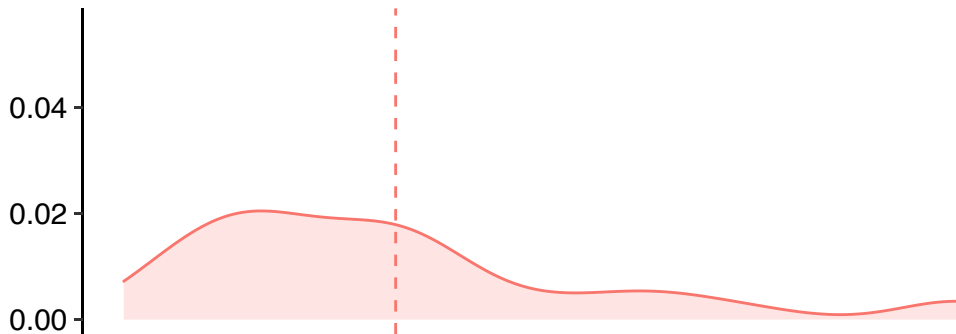

10d

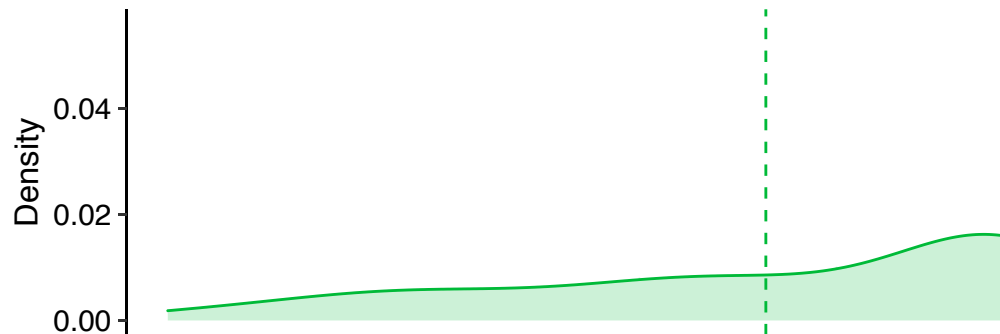

30d

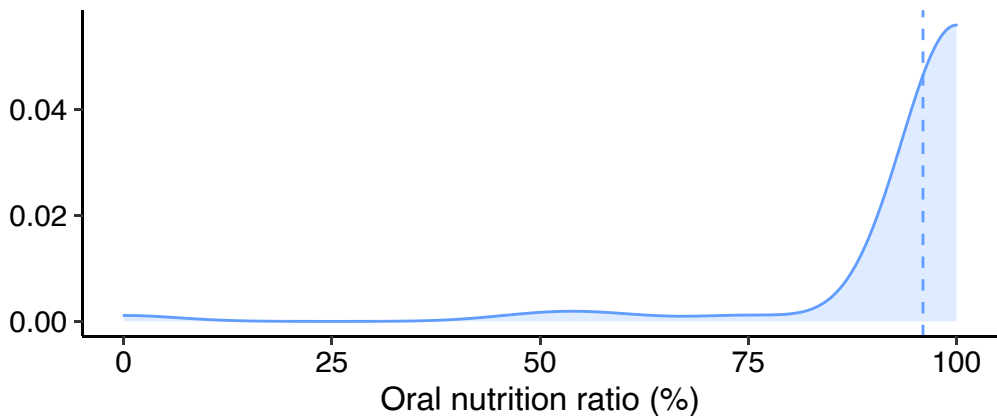

Days of life

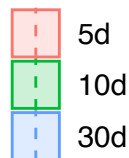

Supplement: Supplemental Material [file KGMI_A_1951113_SM3485.zip › supplementary/downloadFromZipFile3.pdf]

NoP

PRO

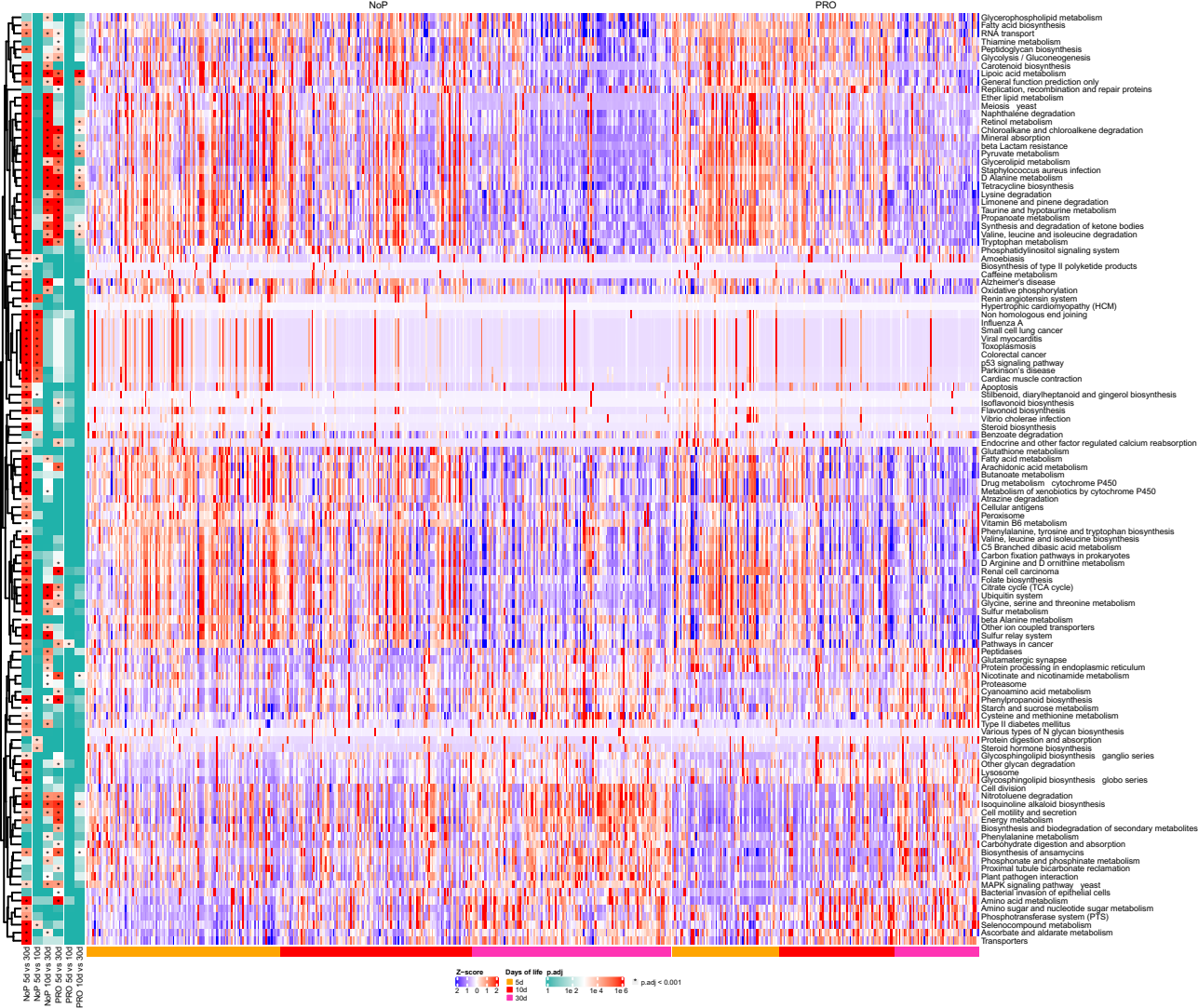

Supplement: Supplemental Material [file KGMI_A_1951113_SM3485.zip › supplementary/downloadFromZipFile4.pdf]

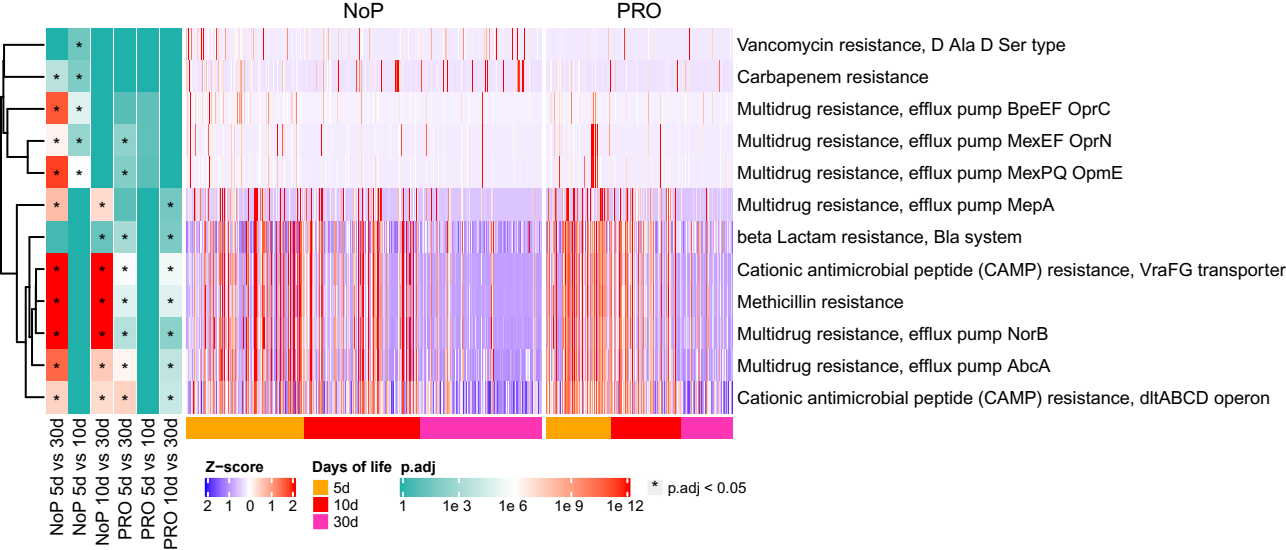

Supplement: Supplemental Material [file KGMI_A_1951113_SM3485.zip › supplementary/downloadFromZipFile5.pdf]

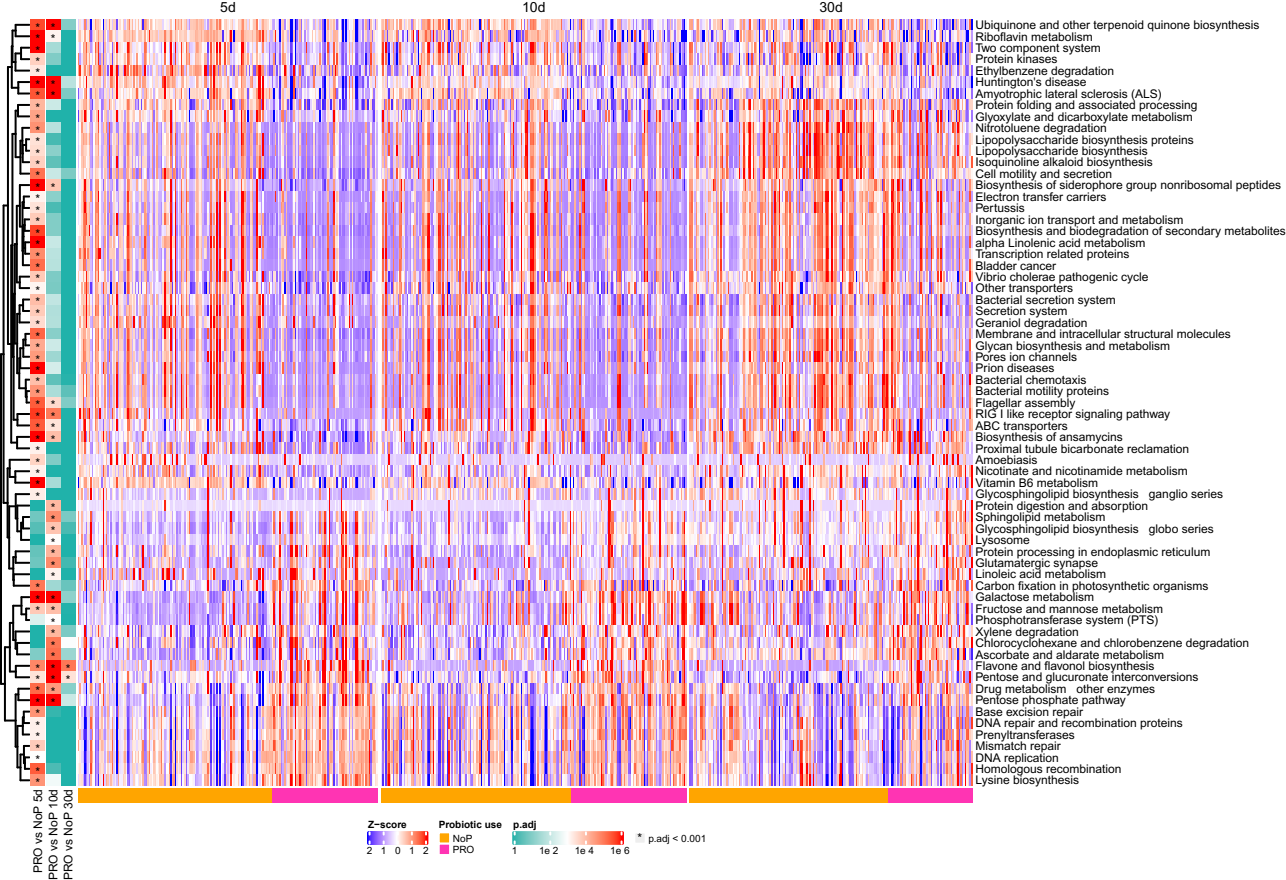

Supplement: Supplemental Material [file KGMI_A_1951113_SM3485.zip › supplementary/downloadFromZipFile6.pdf]
